# Spontaneous Pneumomediastinum, Subcutaneous Emphysema, and Pneumoperitoneum in RT-PCR-Confirmed Measles: A Pediatric Case Report

**DOI:** 10.3390/idr18030039

**Published:** 2026-04-24

**Authors:** Roberto Miguel Damián-Negrete, Alondra Denisse Hernández-Luna, Rocío Guadalupe Cano-Arias, Antonio Durán-Plaza, Judith Carolina De Arcos-Jiménez, Kathya Analí Rodríguez-González, Braulio Dazahel González-Flores, Pedro Iván Navarro-González, Jaime Briseno-Ramírez

**Affiliations:** 1 Department of Pediatrics, Hospital Civil de Guadalajara “Fray Antonio Alcalde”, Guadalajara 44280, Mexico; roberto.damian@cutlajomulco.udg.mx (R.M.D.-N.); alondennys.1996@gmail.com (A.D.H.-L.); canoarias.rocio@gmail.com (R.G.C.-A.); duranplazamcpa@gmail.com (A.D.-P.); kathyrg.24@gmail.com (K.A.R.-G.); gonzalezbd.95@gmail.com (B.D.G.-F.); 2 Centro Universitario de Tlajomulco, Universidad de Guadalajara, Tlajomulco de Zúñiga 45641, Mexico; judith.dearcos@academicos.udg.mx; 3 Department of Pediatrics, Hospital Civil de Oriente, Tonalá 45425, Mexico; pinavarro@hcg.gob.mx

**Keywords:** measles, pneumomediastinum, subcutaneous emphysema, air leak syndrome, Macklin effect, pediatric, case report

## Abstract

Measles remains a major global public health challenge as declining vaccination coverage fuels outbreaks worldwide. Although pneumonia is the most recognized respiratory complication, spontaneous air leak syndrome—including pneumomediastinum, subcutaneous emphysema, and pneumoperitoneum—is rarely documented. We report the case of a 9-year-old previously healthy girl with no documented measles–rubella vaccination who presented with fever, maculopapular exanthem, Koplik spots, and persistent cough. Measles was confirmed by both immunoglobulin M enzyme-linked immunosorbent assay and real-time reverse transcription polymerase chain reaction. She developed sudden cervicothoracic swelling and chest pain. Chest radiography revealed pneumomediastinum and subcutaneous emphysema; computed tomography confirmed extensive air leak including pneumoperitoneum. Flexible bronchoscopy and upper gastrointestinal endoscopy excluded structural airway and esophageal injury. Laboratory evaluation revealed elevated hepatic transaminases, gamma-glutamyl transferase, lactate dehydrogenase, and D-dimer. Conservative management with high-flow supplemental oxygen and clinical surveillance led to progressive resolution. The patient was discharged on hospital day three, asymptomatic and breathing room air. This case highlights the spectrum of air leak complications in measles and supports conservative management in hemodynamically stable pediatric patients when structural injury has been excluded.

## 1. Introduction

Measles is a highly contagious viral infection caused by *Measles morbillivirus*, a member of the genus *Morbillivirus* within the family *Paramyxoviridae* [1,2]. Despite the availability of an effective vaccine, measles has resurged globally, driven by declining vaccination coverage, vaccine hesitancy, and migration [3,4,5]. In 2023 alone, the World Health Organization reported a 20% increase in measles cases and a 43% increase in deaths compared with the previous year, with 57 countries experiencing large or disruptive outbreaks [3,5]. In the Americas, the Pan American Health Organization issued an epidemiological alert in 2025 documenting renewed measles transmission after the region had briefly regained elimination status [6].

Respiratory complications remain the primary cause of measles-associated morbidity and mortality, with pneumonia accounting for the majority of hospitalizations and deaths [1,7]. However, structural complications involving the air leak syndrome spectrum—pneumomediastinum, subcutaneous emphysema, pneumothorax, and pneumoperitoneum—are rarely reported and may be underdiagnosed in epidemic contexts [8,9,10].

Spontaneous pneumomediastinum is defined as the presence of free air within the mediastinum in the absence of trauma, mechanical ventilation, or invasive procedures [11]. The accepted pathophysiological mechanism is the Macklin effect: alveolar rupture secondary to increased intraalveolar pressure gradients, followed by air dissection along bronchovascular sheaths toward the mediastinum [12,13,14]. In the setting of measles, intense cough and bronchioloalveolar inflammation serve as plausible precipitating factors [2,15,16].

The literature on air leak complications in measles is limited, consisting primarily of isolated case reports and small historical series dating back to the 1960s [9,15,16,17]. Recent reports have renewed attention to this entity in the context of current outbreaks [8,18,19].

We present a case of extensive air leak syndrome—including pneumomediastinum, subcutaneous emphysema, and pneumoperitoneum—in a pediatric patient with RT-PCR-confirmed measles, managed conservatively with favorable outcome. This report was prepared in accordance with the CARE (CAse REport) guidelines [20].

## 2. Case Presentation

### 2.1. Clinical History

A 9-year-old previously healthy girl with no reported or documentable measles–rubella vaccination presented to the pediatric emergency department of a tertiary university referral hospital.

Ten days prior to admission, she developed generalized abdominal pain, followed by sore throat and unquantified intermittent fever responsive to paracetamol. A maculopapular exanthem with craniocaudal distribution (beginning on the face and spreading to the trunk and extremities) appeared on illness day 5. Persistent dry, non-productive cough developed subsequently. The mother reported vigorous coughing episodes accompanied by wheezing suggestive of bronchospasm, prompting evaluation by a local physician who prescribed nebulized ambroxol (a mucolytic agent) without clinical improvement. The epidemiological investigation identified travel to Mexico City 12 days before exanthem onset (within the 21-day incubation period) and contact with a symptomatic household member.

Two days before admission, the patient developed sudden subcutaneous swelling of the thorax and face, accompanied by pain in both shoulders and the posterior thoracic region, exacerbated by breathing. She was evaluated at two external facilities before being referred to our institution with suspected subcutaneous emphysema.

### 2.2. Admission Findings

At presentation, the patient was hemodynamically stable. Vital signs were: heart rate 120 bpm, respiratory rate 35 breaths/min, temperature 37.0 °C, blood pressure 90/60 mmHg, and oxygen saturation 95% on room air. Weight was 26 kg and height 126 cm.

Physical examination revealed an alert, reactive child with increased respiratory effort and tachypnea. Palpable crepitation was present from the skull base to the nipple line bilaterally, consistent with extensive subcutaneous emphysema. Auscultation demonstrated preserved vesicular breath sounds with inspiratory crackles in the left hemithorax. The remainder of the examination, including cardiovascular, abdominal, and neurological assessments, was unremarkable. Residual Koplik spots were noted on the buccal mucosa, consistent with the late exanthematous phase of measles.

### 2.3. Diagnostic Workup

#### 2.3.1. Laboratory Findings

Initial laboratory results are summarized in Table 1. The complete blood count showed a normal leukocyte count (4.94 × 10^3^/μL) with unremarkable differential except for an elevated basophil percentage (9.70%, reference 0–2.5%). Hepatic function tests were notably abnormal: gamma-glutamyl transferase (GGT) 109 IU/L (reference 8–30), alanine aminotransferase (ALT) 56 U/L (reference 10–40), aspartate aminotransferase (AST) 64 IU/L (reference 10–50), alkaline phosphatase (ALP) 201 IU/L (reference 53–128), and lactate dehydrogenase (LDH) 734 U/L (reference 91–190). D-dimer was elevated at 1159 ng/mL (reference < 386). Electrolytes, renal function, total bilirubin, albumin, and coagulation parameters (prothrombin time 13.6 s, INR 1.24, activated partial thromboplastin time 32.3 s, fibrinogen 297 mg/dL) were within normal limits or near-normal ranges.

#### 2.3.2. Molecular and Serological Confirmation

On hospital day 1, an oropharyngeal swab was collected, placed in viral universal transport medium, and transported at 2–8 °C under cold-chain conditions to the State Public Health Laboratory. Magnetic bead–based automated nucleic acid extraction was performed, followed by InDRE-validated real-time RT-PCR targeting the measles virus *N* gene and the rubella virus *E1* gene, with human RNase P as an internal control [21]. Results were reported approximately 27 h after specimen collection. Measles virus RNA was detected with a quantification cycle (Cq) value of 24 (positive: ≤40); rubella virus RNA was not detected; and RNase P amplified at Cq 28, confirming specimen adequacy (Figure S1). Serum immunoglobulin M (IgM) enzyme-linked immunosorbent assay (ELISA) was positive with a ratio of 5.401 (positive: >1.100). Rubella virus infection was excluded by both RT-PCR and rubella IgM ELISA. SARS-CoV-2 infection was excluded by a negative reverse transcription polymerase chain reaction test.

#### 2.3.3. Imaging

**Figure 1:**
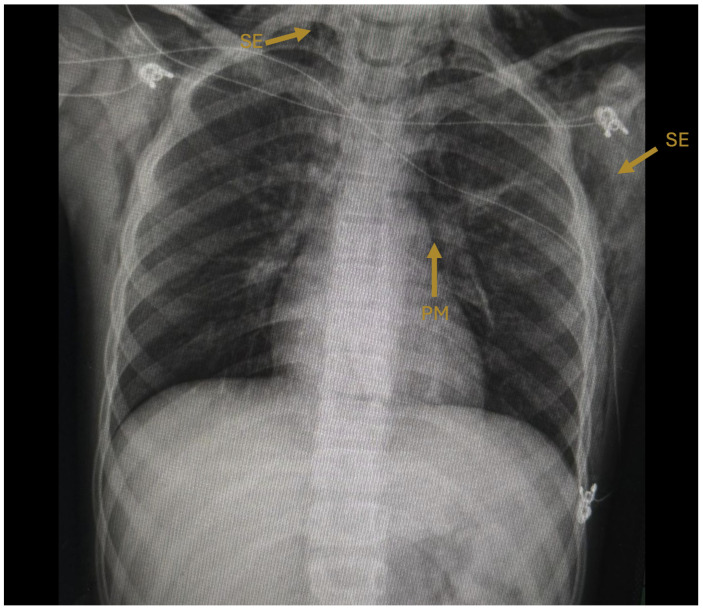
Initial posteroanterior chest radiograph (hospital day 1). Arrows indicate pneumomediastinum (PM), visible as a radiolucent halo outlining the cardiac silhouette, and subcutaneous emphysema (SE) dissecting through the cervical and pectoral soft tissues bilaterally. The trachea is midline and pulmonary parenchyma appears without consolidation.

**Figure 2:**
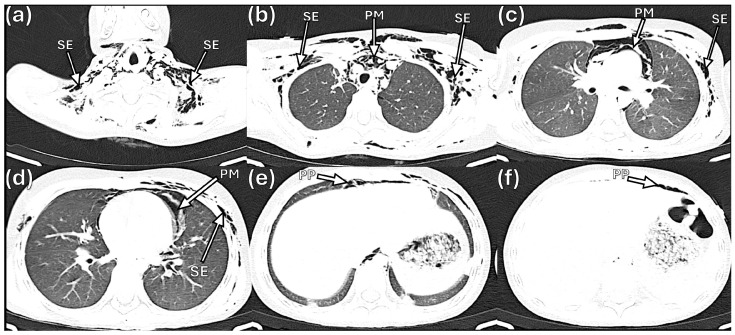
Axial computed tomography of the chest and abdomen (hospital day 2). (**a**) Cervical level (lung window) showing bilateral subcutaneous emphysema (SE) dissecting through the deep cervical soft tissues. (**b**) Upper thorax (lung window) demonstrating pneumomediastinum (PM) with air surrounding the great vessels, and bilateral subcutaneous emphysema (SE) in the pectoral musculature. (**c**) Mid-thorax (lung window) confirming extensive pneumomediastinum (PM) and subcutaneous emphysema (SE). (**d**) Lower thorax (lung window) showing persistent pneumomediastinum (PM) and subcutaneous emphysema (SE) at the level of the pulmonary hila. (**e**) Upper abdomen (soft-tissue window) revealing pneumoperitoneum (PP) as free air anterior to the liver. (**f**) Upper abdomen (soft-tissue window) with additional evidence of pneumoperitoneum (PP) and residual air dissection.

The initial chest radiograph demonstrated pneumomediastinum and extensive subcutaneous emphysema extending from the skull base to the mid-thorax, with a central trachea and normal pulmonary parenchyma (Figure 1). On hospital day 2, computed tomography (CT) of the chest confirmed pneumomediastinum with air dissecting along the esophagus, trachea, and pericardium, as well as pneumoperitoneum and subcutaneous emphysema extending through the cervical, shoulder, and pectoral musculature (Figure 2).

#### 2.3.4. Endoscopic Evaluation

Given the extensive air leak involving multiple compartments, endoscopic evaluation was indicated to exclude structural airway and esophageal injury before attributing the findings to a spontaneous mechanism. Flexible bronchoscopy performed on hospital day 1 under sedation revealed no tracheobronchial lesions or mucosal abnormalities; bronchoalveolar lavage was not performed, as the primary objective was visual exclusion of structural injury. Upper gastrointestinal endoscopy, performed subsequently to exclude esophageal perforation, was similarly normal.

### 2.4. Management and Clinical Course

Diagnosis of air leak syndrome (pneumomediastinum, subcutaneous emphysema, and pneumoperitoneum) secondary to measles infection was established after exclusion of structural airway and esophageal injury.

Conservative management was instituted with supplemental oxygen (initially via nasal cannula at 1 L/min, subsequently escalated to a non-rebreather mask at 10 L/min on recommendation from pediatric surgery to promote nitrogen washout and reabsorption of extrapulmonary air). Intravenous crystalloid maintenance fluids were administered initially and discontinued once oral intake was adequate. No antibiotics or antiviral agents were administered throughout hospitalization. Pediatric surgery and pediatric infectious disease services were consulted. Multiple reassessments by pediatric surgery confirmed no indication for surgical intervention.

**Figure 3:**
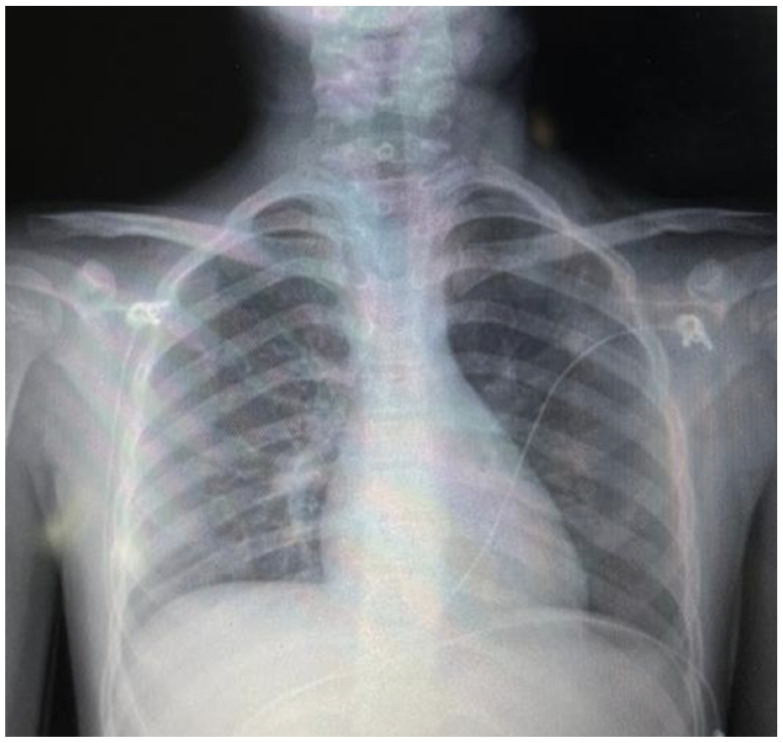
Follow-up posteroanterior chest radiograph (hospital day 3, pre-discharge) demonstrating near-complete resolution of subcutaneous emphysema and pneumomediastinum compared with the admission study (Figure 1). The cardiac and mediastinal contours are now clearly delineated without surrounding radiolucent halos.

The patient remained hemodynamically stable and afebrile throughout hospitalization. Supplemental oxygen was gradually weaned. A follow-up chest radiograph on hospital day 3 demonstrated near-complete resolution of subcutaneous emphysema and pneumomediastinum compared with admission (Figure 3). The patient was discharged on hospital day 3 (7 February 2026), asymptomatic, tolerating a complete oral diet, and maintaining oxygen saturation above 94% on room air. At a scheduled follow-up visit four days after discharge, the patient remained asymptomatic without supplemental oxygen, and repeat laboratory evaluation demonstrated normalization of hepatic enzymes and inflammatory markers (Table 1).

## 3. Discussion

This case illustrates an extensive air leak syndrome—encompassing pneumomediastinum, subcutaneous emphysema, and pneumoperitoneum—as a complication of RT-PCR-confirmed measles in a pediatric patient. Several aspects of this presentation deserve discussion.

### 3.1. Pathophysiology: The Macklin Effect in Measles

The pathogenesis of spontaneous pneumomediastinum is best explained by the Macklin effect [12,14]. This mechanism involves alveolar rupture due to a sudden increase in the intraalveolar pressure gradient—typically provoked by vigorous coughing, bronchospasm, or Valsalva maneuvers—followed by centripetal air dissection along bronchovascular sheaths toward the mediastinum [13,22]. From the mediastinum, air may further dissect into the subcutaneous tissues of the neck and chest, the pleural space (producing pneumothorax), and the retroperitoneal and peritoneal spaces (producing pneumoperitoneum) [13,22,23,24].

In measles, the combination of intense, persistent cough and diffuse bronchioloalveolar inflammation creates favorable conditions for alveolar rupture [2,15,16]. The inflammatory process weakens alveolar wall integrity while paroxysmal coughing generates the pressure gradient necessary for rupture. The presence of three air leak compartments (mediastinum, subcutaneous tissue, and peritoneal cavity) in our patient reflects the extensive and contiguous nature of air dissection along fascial planes.

### 3.2. Epidemiological Context

This case occurred during the ongoing global resurgence of measles, attributed to declining vaccination coverage [3,5,6]. The epidemiological investigation identified an unvaccinated child with a plausible exposure (travel to a metropolitan area during an active outbreak period and contact with a symptomatic household member). The absence of any documented or reported measles–rubella vaccination in a school-age child underscores the consequences of suboptimal immunization coverage. Indeed, measles virus infection has been shown to diminish preexisting antibodies against other pathogens, amplifying the individual and population-level consequences of each missed vaccination opportunity [25].

Data from previous outbreaks suggest that subcutaneous emphysema in measles may not be as rare as traditionally believed. As reviewed by Kaba et al. [8], reported frequencies of subcutaneous emphysema in measles have ranged from 0.59% in a South African observational series [26] to 1.9% in a large pediatric cohort [27] and 3.8% during a refugee-camp outbreak [10]; notably, in that outbreak Moons and Thallinger documented an incidence as high as 15.4% among children under five years in a Somali refugee camp [10]. These figures suggest that air leak complications may be underdiagnosed, particularly in resource-limited settings where imaging is not routinely available.

### 3.3. Hepatic Involvement

Our patient exhibited mild elevations of hepatic enzymes (GGT 3.6× ULN, ALT 1.4× ULN, AST 1.3× ULN, ALP 1.6× ULN) and LDH (3.9× ULN) without hyperbilirubinemia. Measles-associated hepatitis is a recognized complication [4,7,28,29]. In our patient, preserved synthetic function (normal albumin, near-normal coagulation parameters) and complete normalization at follow-up confirm a transient, clinically insignificant hepatic insult consistent with the self-limited hepatitis described in measles, requiring no specific intervention.

The elevated D-dimer (1159 ng/mL, 3× ULN) likely reflects the systemic inflammatory state associated with measles and the tissue damage from air leak rather than a thromboembolic event. Pulmonary embolism was considered clinically unlikely given the patient’s age (9 years), the absence of thromboembolic risk factors, no clinical signs of deep venous thrombosis, and rapid clinical improvement; accordingly, CT pulmonary angiography was not pursued. Ohnishi and Kato demonstrated that intravascular coagulation activation and fibrinolysis occur during the acute febrile phase of measles, providing a pathophysiological basis for D-dimer elevation in this context [30].

### 3.4. Diagnostic Approach

The diagnostic strategy in our case followed a systematic approach to exclude potentially life-threatening etiologies. The differential diagnosis for pneumomediastinum in a pediatric patient includes tracheobronchial rupture, esophageal perforation (Boerhaave syndrome), barotrauma, and foreign body aspiration [11,23]. Flexible bronchoscopy and upper gastrointestinal endoscopy were performed to rule out structural airway and esophageal injury; both were normal, supporting the diagnosis of spontaneous air leak.

Of note, the pneumomediastinum was identified on the initial chest radiograph at admission, before any clinical deterioration, consistent with the observation by Bloch and Vardy that this complication may be radiographically apparent before it is clinically suspected [15]. This finding underscores the importance of careful radiographic interpretation in patients presenting with measles and respiratory symptoms.

### 3.5. Conservative Management

Our management approach—high-flow supplemental oxygen therapy and close surveillance—aligns with current evidence supporting conservative treatment of spontaneous pneumomediastinum in hemodynamically stable patients [31,32,33]. High-flow oxygen promotes nitrogen washout from extrapulmonary air collections, accelerating reabsorption [23,34]. In our patient, the escalation from nasal cannula (1 L/min) to a non-rebreather mask (10 L/min) was recommended by the pediatric surgery team specifically for this purpose.

No antibiotics or antiviral agents were administered during hospitalization. The patient remained afebrile throughout, and the absence of leukocytosis or radiographic consolidation argued against bacterial superinfection. Resolution was achieved within three hospital days, consistent with the benign, self-limited course described in pediatric series of spontaneous pneumomediastinum [11,17,31,33].

Takada et al. have argued against routine invasive evaluation in clinically stable patients with spontaneous pneumomediastinum, noting that many patients undergo unnecessary procedures without altering management [31]. In our case, the extensive subcutaneous emphysema and the context of an active infectious process warranted endoscopic evaluation to exclude structural injury. Once this was excluded, conservative management was both justified and successful.

### 3.6. Comparison with Published Cases

Our case shares similarities with the recent case report and literature review by Kaba et al. [8], who described a 12-year-old unvaccinated male with measles-associated pneumomediastinum and subcutaneous emphysema, confirmed by IgM and PCR, who required pediatric intensive care unit admission with non-invasive respiratory support for three days and received empirical ceftriaxone and vitamin A, with complete resolution of subcutaneous emphysema by day 13. Our case adds several elements: (1) documentation of pneumoperitoneum in addition to pneumomediastinum and subcutaneous emphysema; (2) normal bronchoscopy and upper gastrointestinal endoscopy formally excluding structural injury; (3) characterization of hepatic enzyme elevations as a concurrent finding; and (4) a shorter hospital course (3 days vs. PICU admission with respiratory support).

Mastroianni et al. recently described a 19-year-old female with pneumomediastinum, subcutaneous emphysema, pneumopericardium, and pneumothorax complicating measles; their literature review found no previous reports with all four air leak manifestations combined [18]. Our case adds pneumoperitoneum as an additional compartment, contributing further documentation of the spectrum of air leak complications in pediatric measles. Janmohamed et al. reported pneumomediastinum complicating measles in a vaccinated adult, demonstrating that this complication may occur regardless of prior vaccination status [19].

The historical cases described by Bloch and Vardy [15] in 1968, the pediatric series by McSweeney and Stempel [17] in 1973, and the Nigerian series by Odita and Akamaguna [9] in 1984 established the association between measles and air leak syndrome. Moons and Thallinger reported a high incidence of subcutaneous emphysema during a measles outbreak in a Somali refugee camp, suggesting this complication may be more common than previously recognized in epidemic settings [10]. Piastra et al. reported a clinical series from a pediatric intensive care unit during the 2006–2007 Rome outbreak, in which an index infant presented with measles-induced air leak and acute respiratory distress syndrome requiring aggressive ventilatory and hemodynamic support [16]. Our case updates this association under contemporary diagnostic standards, including molecular confirmation by RT-PCR, CT characterization, and endoscopic exclusion of structural injury.

### 3.7. Limitations

As a single case report, this study cannot establish a causal relationship between measles and the air leak syndrome. However, the temporal association, the compatible pathophysiological mechanism, and the systematic exclusion of alternative etiologies strongly support this link. Although hepatic enzymes normalized at follow-up, serial measurements during hospitalization were not obtained to document the precise time course of hepatic recovery.

## 4. Conclusions

Spontaneous pneumomediastinum, subcutaneous emphysema, and pneumoperitoneum may occur as complications of measles through the Macklin effect, driven by intense cough and bronchioloalveolar inflammation. This case demonstrates that even extensive multi-compartment air leak can follow an uncomplicated, self-limited clinical course with rapid resolution under conservative management. Early radiographic recognition, systematic exclusion of structural airway and esophageal injury, and conservative management with high-flow oxygen therapy constitute a safe and effective approach in hemodynamically stable pediatric patients. In the context of the global resurgence of measles, clinicians should maintain awareness of these rare but clinically significant air leak complications to avoid unnecessary invasive interventions and optimize patient outcomes.

## Figures and Tables

**Table 1:** Laboratory findings at admission (hospital day 1) and at follow-up (day 7 post-discharge).

Parameter	Admission	Follow-Up	Reference Range	Units
Complete blood count				
White blood cells	4.94	6.82	4.60–10.20	×10^3^/μL
Neutrophils	50.63	54.10	37.0–80.0	%
Lymphocytes	23.14	34.20	10.0–50.0	%
Basophils	9.70	0.40	0.00–2.50	%
Hemoglobin	14.31	13.90	12.20–18.10	g/dL
Platelets	238.5	275.0	142.0–424.0	×10^3^/μL
Metabolic panel				
Glucose	84	92	60–125	mg/dL
Creatinine	0.5	0.5	0.5–1.2	mg/dL
Sodium	136	140	135–145	mmol/L
Potassium	3.90	4.20	3.50–5.10	mmol/L
Hepatic function				
GGT	109	22	8–30	IU/L
ALT	56	25	10–40	U/L
AST	64	30	10–50	IU/L
ALP	201	115	53–128	IU/L
LDH	734	172	91–190	U/L
Total bilirubin	0.4	0.5	0.2–1.2	mg/dL
Albumin	4.40	4.30	3.50–5.00	g/dL
Coagulation				
Prothrombin time	13.6	12.1	9–13	s
INR	1.24	1.05	—	—
aPTT	32.3	29.8	25–35	s
Fibrinogen	297	315	200–400	mg/dL
Inflammatory marker				
D-dimer	1159	195	<386	ng/mL

GGT, gamma-glutamyl transferase; ALT, alanine aminotransferase; AST, aspartate aminotransferase; ALP, alkaline phosphatase; LDH, lactate dehydrogenase; INR, international normalized ratio; aPTT, activated partial thromboplastin time.

## Data Availability

Data supporting the findings of this case report are contained within the article. Additional clinical data are not publicly available due to patient privacy restrictions.

## Supplementary Materials

The following supporting information can be downloaded at: https://www.mdpi.com/article/10.3390/idr18030039/s1, Figure S1: Real-time RT-PCR amplification plot (ΔRn versus cycle number). The purple curve (MeV) corresponds to the measles virus *N* gene (Cq = 24), the green curve (RP) represents the human RNase P internal control (Cq = 28), and the orange curve (RuV) shows the rubella virus *E1* gene target (not detected). The positive amplification of the measles *N* gene confirms active viral infection, while RNase P amplification validates specimen adequacy.

## Abbreviations

The following abbreviations are used in this manuscript:

ALP	Alkaline phosphatase
ALT	Alanine aminotransferase
aPTT	Activated partial thromboplastin time
AST	Aspartate aminotransferase
CARE	CAse REport
Cq	Quantification cycle
CT	Computed tomography
ELISA	Enzyme-linked immunosorbent assay
GGT	Gamma-glutamyl transferase
IgM	Immunoglobulin M
INR	International normalized ratio
LDH	Lactate dehydrogenase
RT-PCR	Reverse transcription polymerase chain reaction
ULN	Upper limit of normal
WHO	World Health Organization
