# Supplementary material for: Spontaneous Pneumomediastinum, Subcutaneous Emphysema, and Pneumoperitoneum in RT-PCR-Confirmed Measles: A Pediatric Case Report
Source: Infect Dis Rep. 2026 Apr 24;18(3):39. doi: 10.3390/idr18030039 (PMC13214823; doi:10.3390/idr18030039)
Supplement: Supplementary file 1 [file idr-18-00039-s001.zip › idr-4212285-supplementary.pdf]

Supplementary Materials

Spontaneous Pneumomediastinum, Subcutaneous Emphysema, and Pneumoperitoneum in RT-PCR-Confirmed Measles: A Pediatric Case Report

---

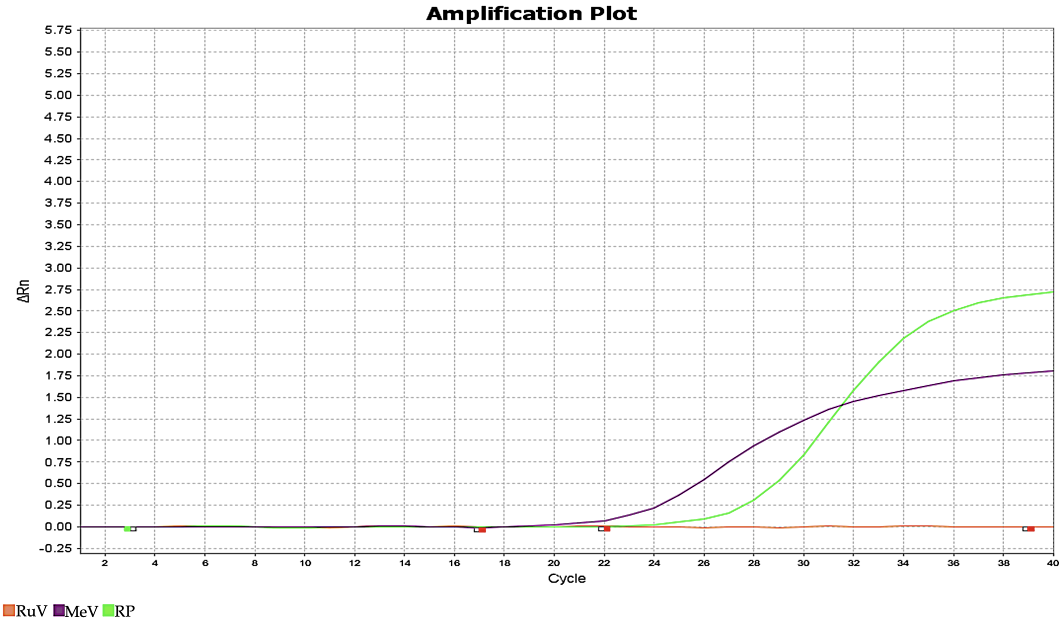

Figure S1. Real-time RT-PCR amplification plot (ΔRn versus cycle number). The purple curve (MeV) corresponds to the measles virus *N* gene (Cq = 24), the green curve (RP) represents the human RNase P internal control (Cq = 28), and the orange curve (RuV) shows the rubella virus *E1* gene target (not detected). The positive amplification of the measles *N* gene confirms active viral infection, while RNase P amplification validates specimen adequacy.
